# ARFID Triggered by Dysgeusia: A Case Report on Light Therapy, Nutritional Support, and Mirtazapine

**DOI:** 10.1002/ccr3.73380

**Published:** 2026-08-20

**Authors:** Aanika Kashyap, Tobi Abibat Saliu, Carrie J. McAdams

**Affiliations:** ^1^ Department of Psychiatry University of Texas at Southwestern Medical Center Texas USA

**Keywords:** case report, circadian rhythm, dysgeusia, eating disorder, functional neurological disorder

## Abstract

Here, we describe an older man who developed avoidant‐restrictive food intake after an acute onset of dysgeusia that persisted for nearly two years. After initiating a multifactorial treatment approach that included light therapy, nutritional support, and mirtazapine, both the eating disorder and dysgeusia resolved within months.

## 1 Introduction

Distorted taste perception, or dysgeusia, is a common yet underrecognized sensory disturbance that affects ~5% of the population and is often idiopathic [1, 2]. In isolation, altered taste perception suggests a functional neurological symptom disorder (FND), but avoidant/restrictive food intake disorder (ARFID) may be a more appropriate diagnosis when dysgeusia is accompanied by persistent food aversion, weight loss, and nutritional deficiencies [3, 4]. Disruption in top‐down modulation of sensory input, including circadian influences on appetite and chemosensory pathways, may contribute to such cases [5, 6]. We describe a patient with profound dysgeusia and malnutrition who experienced rapid clinical recovery following circadian‐based light therapy, mirtazapine, and targeted nutritional support.

## 2 Case History

### 2.1 Presentation

A Caucasian man in his late 60s with no reported past psychiatric history presented with an abrupt onset of dysgeusia, reporting that the first bite of food tasted normal but the flavor of the food would change to rotting meat soon after he began to chew. He first noticed this with a favorite candy. Within two days, all food was intolerable, causing gagging within a few seconds of starting to eat.

The patient first sought treatment with his primary care provider 2 months after the onset of the dysgeusia. Medical history included gout, arthritis, Type 2 diabetes, hyperlipidemia, hypertension, and gastroesophageal reflux disease. He was up‐to‐date on vaccines, had received his fourth booster for the Pfizer COVID‐19 series about one month prior to the dysgeusia, and did not believe he had ever had COVID‐19. At that time, his body mass index (BMI) was 44.9 kg/m^2^. The initial lab workup was normal, and he was encouraged to continue trialing a variety of foods with an expectation that dysgeusia would resolve on its own.

He returned 16 months after symptom onset, having lost approximately 28 kg (BMI 36.8 kg/m^2^). At that time, he was referred to a dietitian. He began intermittent use of nutritional supplement shakes and was told to keep trying different foods. He continued to lose weight and began having diarrhea daily.

One month later, 17 months after onset, the patient underwent a comprehensive diagnostic workup due to the severity and persistence of his dysgeusia and weight loss. Laboratory investigations were notable for a transient vitamin B12 deficiency. Intramuscular injections for vitamin B12 deficiency were given, but no improvement in dysgeusia was noted, and weight loss continued. Zinc and other micronutrient levels were within normal limits. An MRI showed no abnormalities. A needle EMG biopsy did reveal moderate‐to‐severe distal symmetric sensory motor polyneuropathy, a diagnosis attributed to diabetes by the neurologist. Gastroenterological evaluation, including upper endoscopy, ruled out any structural or inflammatory causes of his symptoms. Otolaryngology assessment found no lesions or dysfunction in the oral cavity or upper aerodigestive tract, and formal taste function testing did not demonstrate any peripheral taste bud pathology. Nutritional assessment confirmed severe malnutrition with weakness and muscle wasting. No etiology for dysgeusia or food aversion was identified.

By 20 months after symptom onset, the patient had lost approximately 60 kg (BMI 27.9 kg/m^2^). He was referred to a pain management specialist and received a stellate ganglion block. Symptoms persisted. He requested a referral to an eating disorder clinic and was seen approximately 22 months after onset. At his initial visit, his BMI was 26.6 kg/m2, a loss of 65 kg since initial onset. At that visit, he was using a cane due to weakness related to both the weight loss and underlying gout. He denied symptoms of depression, including sadness, guilt, poor concentration, low self‐esteem, anhedonia, or suicidal ideation.

### 2.2 Differential Diagnosis

Diagnoses considered on presentation to the psychiatry outpatient clinic included neurological disorders, eating disorders, and mood disorders.

From a neurological perspective, neuroimaging, history, and physical exam had not identified any organic problems. Temporal lobe epilepsy can present with gustatory hallucinations, where an abrupt onset of a phantom taste (at times foul) is a well‐recognized phenomenon [7]. However, given the absence of other neurological signs, the dysgeusia was deemed idiopathic, with no metabolic, infectious, or iatrogenic cause identified. This highlights the challenge of identifying organic substrates contributing to functional taste disorders. FND is the most common diagnosis associated with idiopathic dysgeusia. FND would have been the appropriate diagnosis had the patient presented earlier in the course of illness. However, by the time of psychiatric evaluation, more than a year after symptom onset, he exhibited significant nutritional deficiencies, persistent difficulty eating, and had experienced substantial weight loss. Resolution of dysgeusia, an FND symptom, would be unlikely to fully correct the severe disturbance in eating pattern that had developed over the prior year.

As such, his presentation met criteria for avoidant/restrictive food intake disorder (ARFID), classified under the DSM‐5 category of Feeding and Eating Disorders. ARFID is categorized by severe restrictive food intake without the concerns about body weight or shape that occur in anorexia nervosa. For an ARFID diagnosis in the context of a comorbid medical condition, such as dysgeusia here, symptoms observed should also be disproportionate and separable from the comorbid condition. This was seen with the intrusion of the eating problems in his daily life. These eating‐related issues included cognitive ruminations about his health and ability to eat, as well as the physical symptoms related to weight loss.

Mood disorders were also considered, as these can contribute to appetite changes, taste disturbances, and weight loss. At intake, the patient did not endorse sadness or anhedonia, only frustration related to the inability to eat. While there were secondary symptoms of depression endorsed, all were expected sequelae of malnutrition (weight loss, fatigue, concentration, appetite changes). No mood or other mental health symptoms were reported prior to onset of the malnutrition, suggesting that neither the dysgeusia nor the eating problems were sequelae related to a mood disorder.

### 2.3 Treatment Approach

A multimodal treatment approach was immediately initiated to address both central and behavioral components commonly deployed for restrictive eating disorders [8]. First, education on circadian rhythm, appetite regulation, and metabolism was provided [9]. Second, 60 min of morning exposure to a bright light therapy lamp (10,000 lx) was introduced as an intervention targeting the circadian rhythm [10]. Third, mirtazapine was prescribed to reduce nausea and anxiety during his attempts at eating, with a slow titration. His treatment plan and eating responses rapidly improved over the next six months (Table 1).

**TABLE 1:** Timeline of clinical symptoms and treatment plan.

Time point	Clinical symptoms			Multimodal treatment plan		
	Dysgeusia	Eating ability	BMI	Eating guidelines	Light^a^	Mirtazapine
Intake	Severe, everything “Rotten Meat”	Occasional protein shake, nothing else	26.6	5–6 protein shakes and try to eat foods at same times each day	Y	7.5 mg
7 days	Mostly rotten, some tolerated	4–5 shakes, eating some foods daily	26.8	As above	Y	15 mg
1 month	Less rotten, some bitter or bland	3–4 shakes and 2–3 meals daily	28	As above	Y	15 mg
4 months	Variable, bland,or aversive	1–2 shakes and 2–3 meals daily	26.5	If unable to eat meal, use supplement	Y	22.5 mg
5 months	Bland and rarely aversive	As above	27.3	As above	Y	30 mg
7 months	Most foods taste bland but not bad	Regular meals tolerated, no shakes	27.9	As above	Y	30 mg
10 months	As above	Eating 3 normal meals and snacks	30.2	As above	Y	30 mg
13 months	Can now taste spices and sweets	As above	29.5	As above	Y	15 mg^b^
17 months	Sweets do not always stay sweet	As above	30.8	As above	Y	15 mg
20 months	As above	As above	30.2	As above	Y	15 mg

^a^ Light therapy prescribed as 10,000 lx indirect exposure for 30–60 min every morning at the same time.

^b^ Dose reduced due to resolution of the eating as well as mood symptoms.

Within a week of initiating these interventions, the patient reported a significant reduction in the aversive dysgeusia and began to eat solid foods. He reported a month later that although his sense of taste was still abnormal, instead of an aversive flavor, he was experiencing very little flavor from food. He said, “Now that food tastes like nothing, I can eat nothing”. He was now eating three meals consistently and protein shakes most days. Both the patient and his wife also noted that the patient had improved energy, interest, and overall mood relative to his state for several months prior to the initial appointment. At his initial as well as subsequent visits, the patient endorsed improvement in mood with increased food intake, suggesting that a depressive disorder may have developed in response to the malnutrition and then resolved as nutrition improved.

Over the next year, the patient resumed a varied solid‐food diet and regained strength, no longer requiring a cane. His diarrhea resolved, his energy improved, and weight stabilized. Dysgeusia remained improved, with only mild residual taste changes, most notably reduced sweetness in desserts. Anhedonia resolved, and the secondary depressive symptoms were completely remitted by five months. He was started on gabapentin for persistent arthritic pain. A mixed sensory‐motor neuropathy in his hands remained but had improved from initial presentation. The mirtazapine was maintained at 30 mg daily for one year, then reduced to 15 mg daily. During that year, he regained approximately 13 kg and has now stabilized with a BMI of 30.2 kg/m^2^. He continues daily light therapy, which he finds significantly beneficial for energy. He has not had recurrence of dysgeusia or other ARFID symptoms.

## 3 Discussion

This case highlights a rare presentation of ARFID triggered by severe dysgeusia, both of which were effectively treated by a combination of bright light therapy, mirtazapine, and behavioral interventions related to types and timing of nutritional intake. In considering etiology, the patient's report “The first bite tastes okay, but then it turns into a foul, rotting meat flavor” suggests a central rather than peripheral neurological disorder. Interestingly, the patient experienced the dysgeusia as dynamic, rapidly worsening with chewing after initiating eating. This suggests that the patient's central nervous system began providing this aversive feedback related to taste perception during the eating episodes.

**FIGURE 1:**
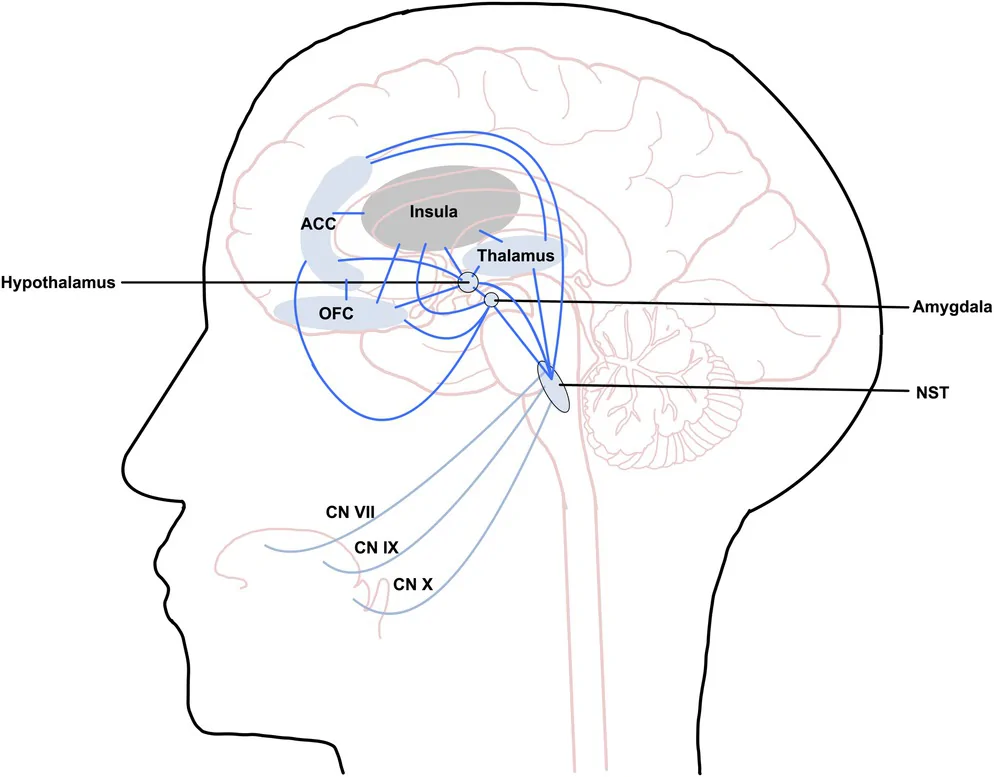
Central pathways of taste perception and integration. Taste perception is initiated by cranial nerves VII, IX, and X, which transmit signals from the peripheral taste receptors in the tongue to the brainstem nucleus of the solitary tract (NST), thalamus, and insula. Central feedback related to food stimuli can include inputs from the orbitofrontal cortex (OFC), which is primarily associated with hedonic valuation, the amygdala, which is associated with aversive learning, and the anterior cingulate cortex (ACC), which is related to emotional and remembered associations. The hypothalamus monitors hunger, satiety, and appetite, which also modulate the value and importance of food.

Taste perception is highly complex, with bidirectional signals in many different neural regions, including areas in the brainstem, thalamus, hypothalamus, and cortical regions (Figure 1). Taste is initiated by cranial nerves VII, IX, and X, which transmit signals from the peripheral taste receptors in the tongue to the brainstem, thalamus, and insular cortex. These signals are further incorporated with olfactory and emotional inputs in the orbitofrontal cortex, where the hedonic value of food is assigned [11]. Within this sensory pathway, the paraventricular nucleus of the thalamus (PVT) is notable for its contribution towards avoidance behaviors due to connections with brain regions associated with fear learning and expression [12]. Taste signaling also engages the hypothalamic circuit, which regulates hunger, food‐seeking behavior, and reward‐based feeding [13, 14]. As signals progress through this network, they are further influenced by multisensory and affective inputs, including sensory neurons involved in aversive sensations like pain and nausea [15]. These cross‐modal interactions may explain how aversive responses may be triggered by functional taste distortions despite intact peripheral signaling. This is consistent with central dysregulation of taste and feeding behavior [15]. There have been multiple prior reports of centrally mediated dysgeusia. While dysgeusia affects around 5% of the population, cases causing malnutrition and psychiatric comorbidities continue to be uncommon and difficult to treat [1]. One documented case describes a patient who developed a persistent taste aversion and weight loss after undergoing an ultrasound thalamotomy for tremor control. The symptoms only resolved following a subsequent thalamic procedure [16]. Another report describes dysgeusia as an early somatic symptom of MDD, highlighting the psychiatric influences on taste processing and experience [17].

Emerging evidence underscores the role of top‐down modulation in shaping sensory experiences, particularly in functional neurological as well as in feeding and eating disorders. Higher‐order cortical regions, like the orbitofrontal cortex and insula, encode expectations and assign value based on prior experiences and contexts [11]. In one recent study of adolescents and young adults with ARFID, low activation of reward circuits and the hypothalamus was observed in response to food with elevated activity in brain regions involved in fear learning, sensory integration, and cognitive control [18]. Expectation‐based errors or beliefs about food are hypothesized to then reinforce avoidance. This framework is consistent with this patient's experience and description of the dysgeusia, which involved a cycle of conditioned aversion to food once its flavor had been registered as “disgusting”.

Viewing the case through the lens of an ARFID diagnosis also guided use of a multidisciplinary approach to treatment focusing on nutritional rehabilitation, mirtazapine, and circadian‐based light therapy [19]. Nutritional rehabilitation is considered the cornerstone of ARFID management [20]. Oral food intake is the preferred method to initiate the refeeding protocol since appetite stimulation and exposures to diminish the food aversions are a key therapeutic goal [21, 22]. Our patient was asked to focus on eating consistently with consideration of timing, types of foods, and amounts. Liquids were encouraged, given the patient's report that his aversion increased with chewing. He began with protein supplements at bedtime and in the morning and set the timing of other meals to be fixed. Prior to working with our team, both the content of food as well as the timing of attempts at eating were erratic.

Importantly, the patient perceived the bright light therapy to have the most immediate impact on his overall eating and energy, enabling him to make the nutritional changes recommended. From a neuroscience preclinical perspective, the suprachiasmatic nucleus (SCN), the body's circadian pacemaker, regulates sleep–wake cycles, appetite rhythms, and hormone secretion [23]. The SCN is located within the hypothalamus, which is also involved in taste coding, hunger signaling, and food‐seeking behaviors [13, 14]. Disruptions in circadian alignment can contribute to changes in appetite, sensory sensitivity, and motivation to eat and are well documented in MDD [24, 25, 26]. Bright light therapy retrains the SCN through timed exposure to bright light and has shown therapeutic value in improving depressive symptoms especially for patients with appetite dysregulation in conjunction with mood symptoms [10, 27]. Clinically, bright light therapy has reduced disordered eating in several small studies and case reports in the literature discussing its use in anorexia nervosa, bulimia nervosa, binge‐eating disorder, and night eating [28, 29]. A biobehavioral circadian model of restrictive and binge eating has been proposed, suggesting that circadian interventions be deployed to facilitate resolution of eating disorders [30]. To our knowledge, bright light therapy has not previously been reported for cases of either ARFID or dysgeusia. In our patient, light therapy likely improved the consistency of appetite, hunger, energy, and satiety signals, while also increasing his motivation and ability to make the eating pattern changes necessary to resolve the ARFID.

Finally, mirtazapine was initiated due to its appetite‐enhancing, mood, and anxiolytic properties. Mirtazapine, sertraline, fluoxetine, olanzapine, and cyproheptadine all have been designated to have a low level of evidence and a weak recommendation for use in ARFID by the World Federation of Societies of Biological Psychiatry task force for eating disorders, with no medications showing stronger evidence or recommendations [31]. The evidence supporting the use of mirtazapine in ARFID derives from two case reports in pediatric patients and a retrospective review of the impact of mirtazapine on ARFID children and young adults in a partial hospital eating disorder program [32, 33, 34]. Another case report has described mirtazapine as an effective treatment for drug‐induced dysgeusia in an elderly patient, with symptoms resolving within one week [35], a finding also reflected in our patient's therapeutic timeline. A final consideration supporting the use of mirtazapine here is that mood and anxiety disorders are commonly comorbid in ARFID [36]. Mirtazapine improves sleep, anxiety, and suicidality in depressed patients in the first one to two weeks of treatment [37, 38].

One limitation of this case is that we cannot establish causality in relation to the development of the initial dysgeusia. Nevertheless, the history and timeline clearly demonstrate that the dysgeusia precipitated the development of ARFID. Another limitation is that we cannot determine exactly which of the interventions provided led to improvements in the dysgeusia and ARFID, as both problems resolved together in response to this multifocal treatment plan.

## 4 Conclusion

In sum, this clinical case illustrates the importance of considering central mechanisms in the etiology of dysgeusia and describes the use of bright‐light therapy in conjunction with meal timing and mirtazapine as key interventions in the restoration of dysregulated eating patterns. Sensory, circadian, and neuropsychiatric factors can all contribute to treating restrictive eating. Light therapy may be an underutilized treatment that is effective for functional neurological disorders as well as feeding and eating disorders.

## 5 Patient's Perspective

I had given up on eating. Everything tasted awful, like metal or bitter chemicals. I was desperate and scared. At first, I thought the vaccine caused it, but doctors couldn't find any connection. No one seemed to take it seriously until I lost a lot of weight and felt awful. It was scary not having answers and not being able to eat. When the light therapy was suggested, I didn't expect much, but in a few days, I noticed things didn't taste as bad. Slowly I started to eat again and feel like myself. I recommend the lamp to everyone because it makes a big difference in my life.

## Author Contributions

**Carrie J. McAdams:** supervision, writing – review and editing, investigation, conceptualization. **Tobi Abibat Saliu:** conceptualization, writing – original draft, writing – review and editing, investigation. **Aanika Kashyap:** conceptualization, writing – original draft, writing – review and editing, visualization.

## Funding

The authors have nothing to report.

## Consent

Written informed consent was obtained from the patient for the publication of this case report and any accompanying clinical images. A copy of the written consent is available for review by the editor‐in‐chief of this journal.

## Conflicts of Interest

The authors declare no conflicts of interest.

## Data Availability

Data sharing not applicable to this article as no datasets were generated or analysed during the current study.
